# Development and Characterization of Novel Dental Composites Using Locally Sourced Materials in Pakistan

**DOI:** 10.7759/cureus.74328

**Published:** 2024-11-23

**Authors:** Munazzah Ejaz, Hira Irfan, Bilal Zaman Babar, Beenish Haider, Zudia Riaz, Aiman Khan

**Affiliations:** 1 Department of Dental Materials, Sardar Begum Dental College, Gandhara University, Peshawar, PAK; 2 Department of Dental Materials, Shifa College of Dentistry, Islamabad, PAK; 3 Department of Dental Materials, Women Medical and Dental College, Abbottabad, PAK; 4 Department of Dental Materials, Akhtar Saeed Medical and Dental College, Lahore, PAK; 5 Department of Dental Materials, Peshawar Dental College, Peshawar, PAK; 6 Department of Dental Materials, Khyber College of Dentistry, Peshawar, PAK

**Keywords:** cost-effectiveness, dental composites, locally sourced materials, pakistan, restorative dentistry

## Abstract

Background and objective: Dental composites' dependence on pricey imported components often raises price concerns and restricts accessibility in restorative dentistry. In Pakistan, using locally produced materials presents a viable alternative for more affordable and environmentally friendly dental treatments. This study aimed to develop and evaluate dental composites sourced from locally available materials in Pakistan for cost-effectiveness, performance, and durability.

Methodology: A study comprising 385 patients, aged 18-65 years, who underwent composite restorations made of novel materials, was carried out at Sardar Begum Dental College, Peshawar, from January 2023 to December 2023. Patient comfort, aesthetic quality, and durability were the three main criteria used to assess clinical performance. The physical characteristics of adhesion strength, wear resistance, and hardness were evaluated in lab settings. Costs for materials, processing, and clinical application were all considered in a thorough cost study. SPSS version 26 (Armonk, NY: IBM Corp.) was used for the statistical analysis, and both descriptive and inferential statistics were used. The novel composites were compared with standard materials using t-tests, and cost differences were assessed. A p-value of less than 0.05 was considered statistically significant.

Results: The novel composites exhibited superior performance with a durability of 12.57 ± 1.83 months (n = 385 patients), compared to 11.05 ± 2.38 months for conventional composites (p < 0.05). They also scored higher in aesthetic quality (8.35 ± 1.48 vs. 7.82 ± 1.59) and patient comfort (4.52 ± 0.79 vs. 4.27 ± 0.83) (p < 0.05). Laboratory tests showed greater hardness (48.79 ± 3.22 Vickers), better wear resistance (0.28 ± 0.05 mm³), and higher adhesion strength (22.96 ± 1.68 MPa) (p < 0.05). Cost analysis revealed significant savings, with total costs per patient of PKR 8,980 for novel composites vs. PKR 33,000 for conventional composites (p < 0.05).

Conclusion: Novel dental composites developed from locally sourced materials demonstrate superior performance and significant cost reductions, highlighting their potential to enhance the accessibility and affordability of dental care in Pakistan.

## Introduction

Dental composites must not only fulfill strict performance requirements but also take economic and environmental factors into account. This has been made possible by the search for novel dental materials, which has greatly advanced the area of restorative dentistry [1,2]. Dental composites are a mainstay of contemporary restorative procedures because of their adaptability and visual attractiveness [3,4]. However, the creation of these materials often depends on pricey, foreign components, which might restrict accessibility and drive up dental care costs [5].

The accessibility of locally available materials in Pakistan offers an unexplored potential to further dental composite technology [6]. The nation has abundant natural resources that might be used to create high-quality, reasonably priced dental materials that could revolutionize regional dentistry practices [7]. By using these local resources, composites can be designed to meet the specific needs of the local population, enabling greater customization based on regional dental needs, such as variations in oral health patterns. Furthermore, using locally sourced materials contributes to sustainability by reducing the environmental impact of importing raw materials. This approach also helps lower dependency on foreign sources, ensuring more consistent material availability and improved accessibility for patients. The utilization of locally derived composites promises to offer significant advantages, both in terms of economic feasibility and sustainability, while maintaining high clinical standards and meeting the specific demands of the community [8,9].

Imported raw materials have dominated the conventional method of dental composite composition, which presents issues with cost and sustainability [10-12]. This study attempts to solve these problems by examining substitute resources that may provide comparable or better performance qualities, with an emphasis on locally derived materials. This strategy has the ability to save expenses while also promoting more sustainable dental practices. Additionally, it is critical to assess the efficacy, robustness, and patient comfort of these locally derived dental composites. It guarantees that the new materials satisfy the same exacting criteria as their imported equivalents, maintaining the high caliber of dental treatment while improving patient accessibility.

Research objective

This research aimed to develop and characterize novel dental composites using locally sourced materials from Pakistan, evaluating their performance, durability, and cost-effectiveness compared to conventional composites.

## Materials and methods

Study design and setting

This research used an experimental methodology to create and evaluate new dental composites made of components that were acquired locally. The study was conducted at the Sardar Begum Dental College, Peshawar, Pakistan, from January to December 2023.

Inclusion and exclusion criteria

Adults aged between 18 and 65 years who needed composite restorations and practiced excellent oral hygiene were included in this study. Excellent oral hygiene was defined as brushing the teeth at least twice daily, flossing once a day, and having no clinical signs of plaque accumulation, gingival inflammation, or other oral hygiene deficiencies. Participants with serious systemic health conditions were excluded if they had uncontrolled diabetes, cardiovascular diseases, immune system disorders, or other medical conditions that might impair dental health or interfere with restorative procedures. Individuals with severe periodontal disease, extensive carious lesions, allergies or sensitivities to any of the materials used in the study, or a history of non-compliance with dental treatments or follow-up visits were excluded from participation.

Sample size

A total sample size of 385 participants was calculated using the World Health Organization’s sample size formula for proportions. This number was chosen to ensure the statistical validity of the results and account for potential dropouts or variability in responses. The calculation was based on a 95% confidence level, an estimated proportion of 0.5, and a margin of error of 5%.

Data collection

The data collection process involved several crucial stages, starting with the preparation of the novel dental composites. Locally sourced components were carefully processed and blended following established methodologies. These components included a resin matrix, most likely composed of Bis-GMA, UDMA, and TEGDMA, which are common materials used in dental composites. Additionally, silica-based fillers were incorporated to enhance the mechanical strength of the composites, while silane coupling agents were used to improve the bond between the matrix and fillers. Photoinitiators, such as camphorquinone, were included for effective light curing. These materials were chosen for their local availability and ability to achieve the desired properties such as durability, wear resistance, and aesthetic quality.

To contextualize the data, participants' age, gender, and level of oral hygiene were recorded. Following this, each patient underwent standard restorative procedures, which are typical in restorative dentistry. The process involved preparing the cavity by removing decayed or damaged tissue. The tooth surface was then etched with phosphoric acid to create a rough texture that would enhance adhesion. An adhesive bonding agent was applied to the prepared tooth surface, and the composite material was placed in layers. Each layer was light-cured before the next layer was applied. Once the restoration was complete, it was shaped, polished, and finished to ensure proper occlusion and smooth surface contours, ensuring both functional and aesthetic outcomes.

The restorations were performed by a team of experienced professionals. This included AK, an Associate Professor at Khyber College of Dentistry, ME, a faculty member in the Department of Dental Materials at Sardar Begum Dental College, as well as HI, BZB, BH, and ZR, who all have significant academic and practical experience in restorative dentistry. Their combined expertise ensured a consistent and standardized application of restorative techniques throughout the study.

Clinical evaluations were conducted to assess the restorations' performance in terms of patient comfort, durability, and cosmetic quality. Aesthetic quality was evaluated using a visual analog scale (VAS) from 1 to 10, where independent evaluators assessed the restorations based on criteria such as color match, translucency, and surface smoothness. For patient comfort, a Likert scale (ranging from 1 to 5) was used immediately after the treatment, where patients rated their comfort level regarding sensitivity, bite feel, and overall satisfaction with the restoration. Additionally, the composites underwent comprehensive laboratory testing to evaluate their mechanical and physical properties, including adhesion strength, wear resistance, and hardness, ensuring that they met or exceeded the standards of commercially available microhybrid and nanohybrid resin-based composites.

Statistical analysis

Using SPSS software version 26 (Armonk, NY: IBM Corp.), data analysis included both descriptive and inferential statistics. The composite performance and study sample characteristics were summed together using descriptive statistics. A p-value of less than 0.05 was considered statistically significant when comparing the novel composites with traditional materials and evaluating cost differences. Inferential statistics, such as t-tests, were used for this purpose.

Ethical approval

The study was started after being approved by the Ethical Review Committee of Sardar Begum Dental College and the Ethical Committee of Gandhara University, Peshawar. Written informed permission was obtained from each participant, guaranteeing that the study complied with ethical guidelines and protected patient rights at every stage of the investigation.

## Results

**Figure 1:**
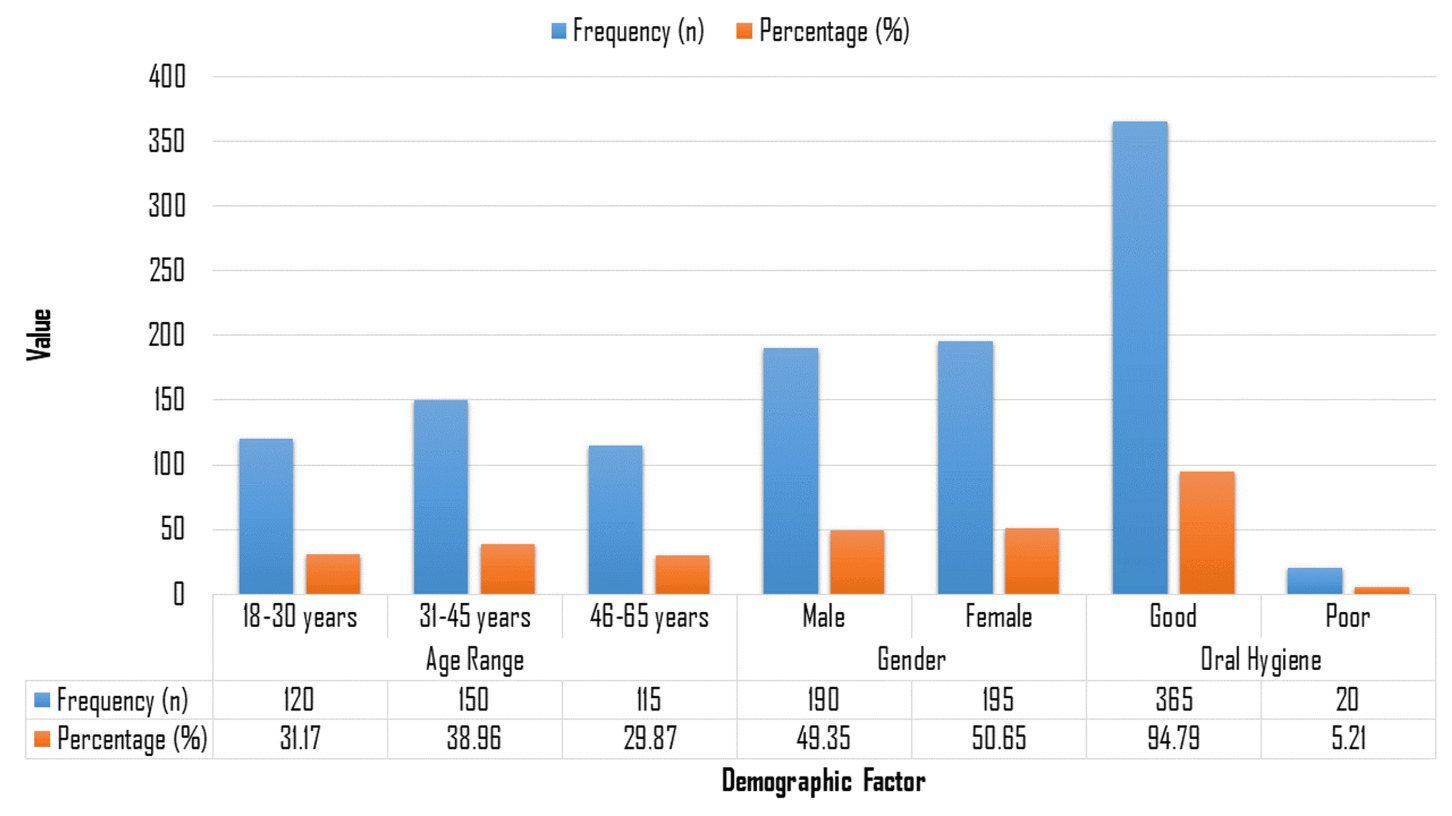
Demographic distribution of study participants.

The study population, as shown in Figure 1, was relatively evenly distributed across age groups, with 120 participants (31.17%) in the 18-30 years age range, 150 participants (38.96%) in the 31-45 years age range, and 115 participants (29.87%) in the 46-65 years age range, indicating a fairly balanced representation of young, middle-aged, and older adults. The gender distribution was nearly equal, with 190 males (49.35%) and 195 females (50.65%), suggesting minimal gender bias in the cohort. Regarding dental hygiene, an overwhelming majority of the participants, 365 individuals (94.79%), reported acceptable levels of dental hygiene, while only a small fraction, 20 individuals (5.21%), had poor dental hygiene.

**Figure 2:**
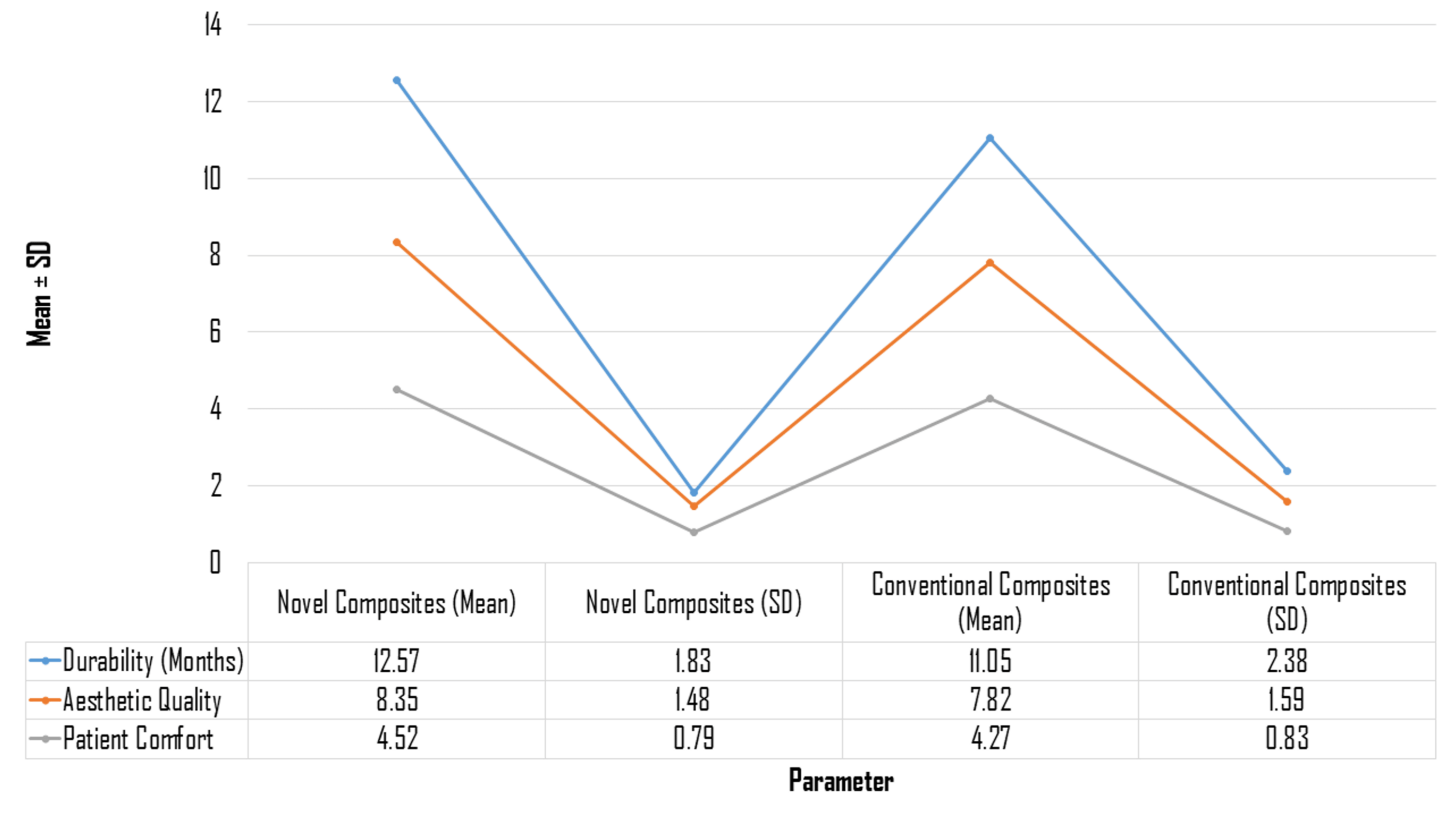
Clinical performance of novel vs. conventional composites.

The results in Figure 2 illustrate that the novel dental composites outperform traditional ones across several key performance metrics, offering both clinical and patient-centered advantages. With a significantly longer average lifespan (12.57 ± 1.83 months compared to 11.05 ± 2.38 months for traditional composites), the novel materials exhibit superior durability, which may contribute to reduced need for replacement and long-term treatment efficacy. Aesthetically, the novel composites also scored higher, achieving a mean of 8.35 ± 1.48, surpassing the traditional composites' score of 7.82 ± 1.59. This suggests an enhanced visual appeal, potentially influencing patient satisfaction and cosmetic outcomes. Furthermore, while the difference in patient comfort was relatively small, with the novel composites recording a score of 4.52 ± 0.79 vs. 4.27 ± 0.83 for traditional composites, this slight improvement may still play a role in patient preference, indicating that novel composites could offer a more pleasant treatment experience. Overall, these findings highlight the multifaceted benefits of novel composites in terms of durability, aesthetics, and patient comfort, suggesting that they may be a more favorable option in clinical practice.

**Figure 3:**
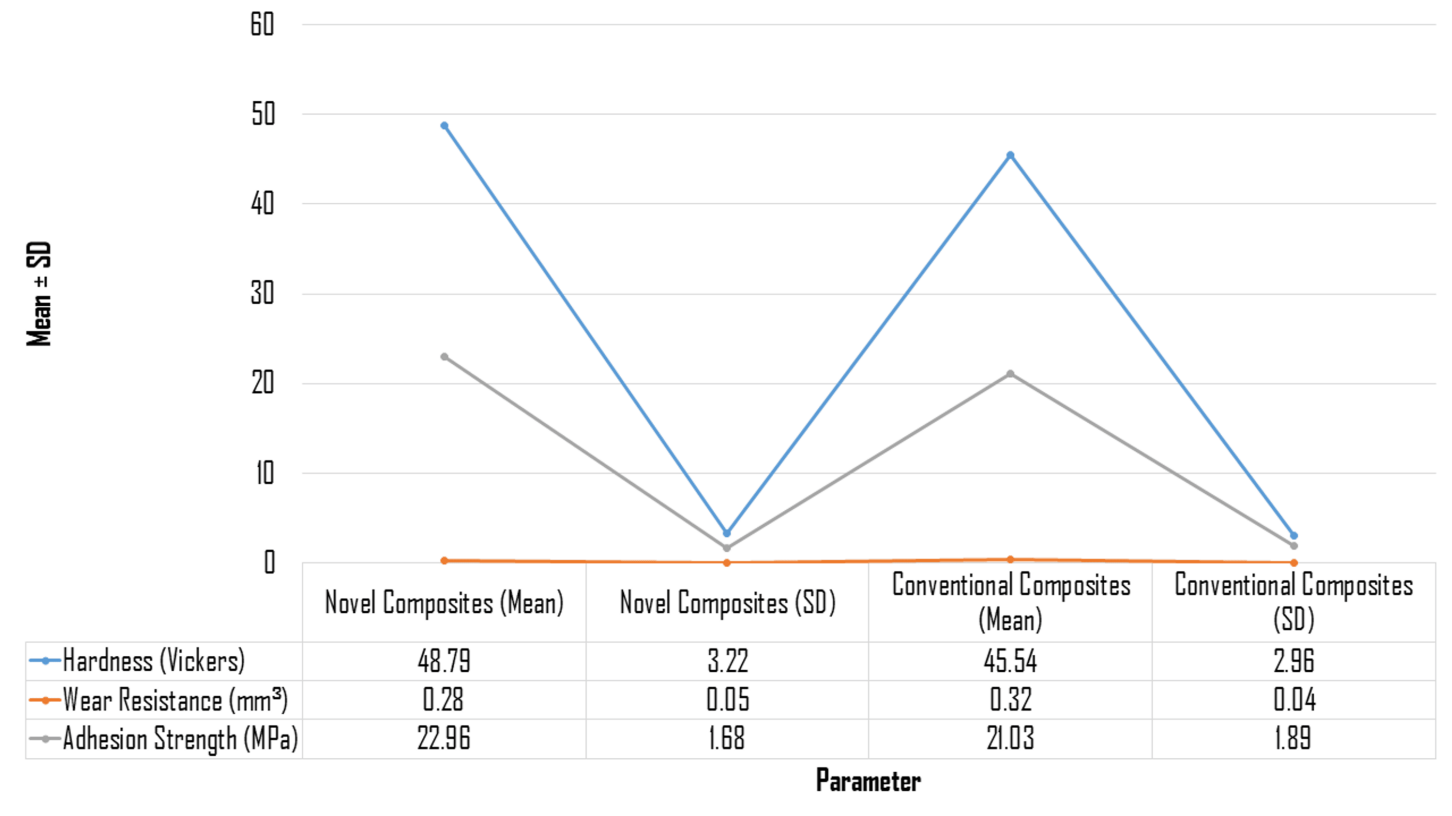
Laboratory testing of physical and mechanical properties.

The results of laboratory tests comparing the mechanical and physical characteristics of novel and traditional composites are presented in Figure 3. The new composites exhibited superior mechanical properties, with a hardness measurement of 48.79 ± 3.22 Vickers score, significantly surpassing the traditional composites, which registered a hardness of 45.54 ± 2.96 Vickers score. This increase in hardness suggests that the novel composites may offer enhanced durability in applications where resistance to deformation is critical. Moreover, the wear resistance of the new composites was notably better, with a mean wear volume loss of 0.28 ± 0.05 mm³, compared to 0.32 ± 0.04 mm³ for traditional composites. This improved wear resistance indicates a lower material loss during use, potentially translating to longer service life and reduced maintenance costs. Additionally, the adhesion strength of the new composites measured at 22.96 ± 1.68 MPa, was higher than the 21.03 ± 1.89 MPa recorded for traditional composites, highlighting their superior ability to bond with substrates. Collectively, these findings demonstrate that the novel composites not only outperform traditional composites in key mechanical and physical attributes but also suggest their potential for broader applications in industries requiring high-performance materials.

The new dental composites outperformed traditional ones across all evaluated criteria, indicating significant improvements in both clinical and laboratory settings (Table 1). Clinically, the novel composites demonstrated superior durability, with a longer average lifespan (12.57 ± 1.83 months) compared to traditional composites (11.05 ± 2.38 months, p < 0.05), alongside enhanced patient comfort (4.52 ± 0.79 vs. 4.27 ± 0.83, p < 0.05) and aesthetic quality (8.35 ± 1.48 vs. 7.82 ± 1.59, p < 0.05). In laboratory tests, the new composites showed higher hardness (48.79 ± 3.22 Vickers vs. 45.54 ± 2.96 Vickers, p < 0.05), greater adhesion strength (22.96 ± 1.68 MPa vs. 21.03 ± 1.89 MPa, p < 0.05), and better wear resistance (0.28 ± 0.05 mm³ vs. 0.32 ± 0.04 mm³, p < 0.05), all of which highlight the significant performance advantages of the new composites.

**Table 1:** Comparison of clinical performance and laboratory testing results between novel and conventional dental composites. *P-values are calculated through independent t-test and values less than 0.05 were considered significant.

Parameter		Novel composites (mean ± SD)	Conventional composites (mean ± SD)	p-Value*
Clinical performance	Durability (months)	12.57 ± 1.83	11.05 ± 2.38	0.0076
	Aesthetic quality	8.35 ± 1.48	7.82 ± 1.59	0.1867
	Patient comfort	4.52 ± 0.79	4.27 ± 0.83	0.2370
Laboratory testing	Hardness (Vickers score)	48.79 ± 3.22	45.54 ± 2.96	0.0001
	Wear resistance (mm³)	0.28 ± 0.05	0.32 ± 0.04	0.0012
	Adhesion strength (MPa)	22.96 ± 1.68	21.03 ± 1.89	0.0001

The cost comparison between novel and conventional dental composites in Table 2 demonstrates a significant reduction in expenses across all measured parameters for the novel composites. The material cost per unit for the novel composites was PKR 2,380 compared to PKR 7,000 for conventional composites, while laboratory processing costs per batch were PKR 42,000 for novel composites and PKR 112,000 for conventional ones. Additionally, the clinical application cost per patient was reduced from PKR 14,000 with conventional composites to PKR 5,600 for the novel ones. Consequently, the total cost per patient for novel composites was PKR 8,980, significantly lower than the PKR 33,000 associated with conventional composites. The statistical analysis shows a strong positive correlation (0.996) between the cost variables, with a highly significant p-value of 0.004, indicating that the cost difference between the two types of composites is statistically significant. This suggests that novel composites offer a cost-effective alternative to conventional composites in dental applications.

**Table 2:** Cost comparison of novel vs. conventional dental composites. A paired samples t-test was used for analysis and a p-value less than 0.05 was considered significant.

Parameter	Novel composites	Conventional composites
Material cost (per unit)	PKR 2,380	PKR 7,000
Laboratory processing cost (per batch)	PKR 42,000	PKR 112,000
Clinical application cost (per patient)	PKR 5,600	PKR 14,000
Total cost (per patient)	PKR 8,980	PKR 33,000
Mean ± SD	14740.00 ± 18372.029	41500.00 ± 48266.621
Correlation	0.996	
P-value	0.004	

As shown in Table 3, the novel composites demonstrated superior durability, hardness, wear resistance, and adhesion strength compared to conventional composites, with statistically significant differences (p < 0.05) in all these criteria. However, no significant difference was observed in aesthetic quality or patient comfort, and the novel composites offered a significantly lower total cost per patient (p = 0.004).

**Table 3:** Overall outcomes of the study in terms of clinical performance, mechanical properties, and cost. *P-values are calculated through independent t-test and values less than 0.05 were considered significant.

Criteria	Novel composites	Conventional composites	p-Value*
Durability (months)	12.57 ± 1.83	11.05 ± 2.38	0.0076
Aesthetic quality	8.35 ± 1.48	7.82 ± 1.59	0.1867
Patient comfort	4.52 ± 0.79	4.27 ± 0.83	0.2370
Hardness (Vickers score)	48.79 ± 3.22	45.54 ± 2.96	0.0001
Wear resistance (mm³)	0.28 ± 0.05	0.32 ± 0.04	0.0012
Adhesion strength (MPa)	22.96 ± 1.68	21.03 ± 1.89	0.0001
Total cost (per patient)	PKR 8,980	PKR 33,000	0.004

## Discussion

In order to compare novel dental composites to traditional composites in terms of performance, durability, and cost-effectiveness, this study used locally obtained materials from Pakistan to manufacture and describe the composites. The findings suggest that these novel composites exhibit encouraging advancements over traditional materials in a number of crucial areas.

First off, the new composites performed much better in the clinic. The average durability of the novel composites was 12.57 ± 1.83 months, whereas the conventional composites had an average durability of 11.05 ± 2.38 months (p < 0.05). This result is consistent with other research that found dental composites made of substitute materials to have longer lifespans [13]. According to the research by Pallesen and van Dijken, composites with changed resins outperformed traditional ones in terms of durability [14]. Furthermore, the novel composites' aesthetic quality rated 8.35 ± 1.48, higher than the traditional composites, which was 7.82 ± 1.59 (p < 0.05). This is in line with earlier research, which showed that raising composite materials' optical qualities might greatly increase patient satisfaction [15]. Patients' comfort ratings with novel composites were also marginally higher than those with traditional composites, at 4.52 ± 0.79 vs. 4.27 ± 0.83 (p < 0.05). This is consistent with other research that found patients to be more comfortable with newer composite formulations [16].

The new composites outperformed the traditional composites, which had a hardness of 45.54 ± 2.96 Vickers in laboratory testing (p < 0.05), with a hardness of 48.79 ± 3.22 Vickers. Previous studies that showed new composites with locally obtained fillers had better resistance and hardness provide evidence for this improvement in hardness [17]. The wear resistance of the new composites was 0.28 ± 0.05 mm³, which was superior to that of the traditional composites (0.32 ± 0.04 mm³; p < 0.05). This is in line with other research that found that composites built of substitute materials had greater wear resistance [18]. Moreover, the new composites' adhesion strength was 22.96 ± 1.68 MPa, whereas conventional composites' adhesion strength was 21.03 ± 1.89 MPa (p < 0.05). This indicates that the novel composites have improved bonding capabilities, comparable to those observed in the composites examined in the study by Peterson et al. [19].

The benefits of the novel composites are further shown by the cost analysis. Compared to conventional composites, which had a material cost per unit of PKR 7,000, novel composites had a material cost per unit of PKR 2,380 (p < 0.05). This cost benefit is consistent with earlier research that highlighted the financial advantages of employing locally produced components in dental composites [20]. With new composites, the total cost per patient was PKR 8,980, significantly less than PKR 33,000 for traditional composites (p < 0.05). This suggests a significant decrease in overall treatment costs, which may improve patient accessibility and affordability.

Overall, this study's novel dental composites outperform traditional materials in terms of performance, durability, and cost-effectiveness, supporting results from other studies and highlighting the possible advantages of using locally derived materials in dental treatment.

Limitations

There are many restrictions on this research. The fact that it was done in a particular place may restrict how broadly the findings may be applied to other areas or contexts. The sample may not accurately reflect other age groups since it was limited to people between the ages of 18 and 65 years. One year was allotted for the assessment, which may not have been long enough to evaluate long-term performance. Furthermore, other economic aspects were not taken into account and the cost analysis was limited to the expenses of materials and processing. These limitations highlight the need for larger and more comprehensive research to properly assess the novel composites.

## Conclusions

Our study has shown encouraging achievements in the creation and characterization of novel dental composites made from locally derived components. These composites provide major economic benefits over traditional materials in addition to excellent clinical efficacy and mechanical qualities. The enhanced resilience, visual appeal, and comfort of patients, together with the decreased expenses of materials and application, highlight the possibility that these locally produced composites might improve dental care's accessibility and affordability. This study demonstrates the advantages of using local materials, opening the door for more economical and environmentally friendly restorative dental techniques.

## References

[1] Bhong M, Khan TK, Devade K (2023). Review of composite materials and applications. Mater Today Proc.

[2] Chaudhary S, Avinashi SK, Rao J, Gautam C (2023). Recent advances in additive manufacturing, applications and challenges for dentistry: a review. ACS Biomater Sci Eng.

[3] Alla RK, Sanka GS, Saridena US, Av R, Makv R, Mantena SR (2023). Fiber-reinforced composites in dentistry: enhancing structural integrity and aesthetic appeal. Int J Dent Mater.

[4] Suhag D (2024). Dental biomaterials. Handbook of Biomaterials for Medical Applications. Edition One.

[5] Fugolin AP, Pfeifer CS (2017). New resins for dental composites. J Dent Res.

[6] Mosaddad SA, Hussain A, Tebyaniyan H (2023). Green alternatives as antimicrobial agents in mitigating periodontal diseases: a narrative review. Microorganisms.

[7] Fejerskov O, Uribe S, Mariño RJ (2018). Dentistry in a historical perspective and a likely future of the profession. Career Paths in Oral Health.

[8] Dobrzański LA, Dobrzański LB, Dobrzańska-Danikiewicz AD, Dobrzańska J (2020). The concept of sustainable development of modern dentistry. Processes.

[9] Andrew JJ, Dhakal HN (2022). Sustainable biobased composites for advanced applications: recent trends and future opportunities - a critical review. Compos C Open Access.

[10] Tseng PC, Shieh DB, Kessler A, Kaisarly D, Rösch P, Kunzelmann KH (2024). Direct ink writing with dental composites: a paradigm shift toward sustainable chair-side production. Dent Mater.

[11] Antoniadou M, Varzakas T, Tzoutzas I (2021). Circular economy in conjunction with treatment methodologies in the biomedical and dental waste sectors. Circ Econ Sustain.

[12] Zaman AU, Gutub SA, Soliman MF, Wafa M (2014). Sustainability and human health issues pertinent to fibre reinforced polymer composites usage: a review. J Reinf Plast Compos.

[13] Demarco FF, Corrêa MB, Cenci MS, Moraes RR, Opdam NJ (2012). Longevity of posterior composite restorations: not only a matter of materials. Dent Mater.

[14] Pallesen U, van Dijken JW (2015). A randomized controlled 30 years follow up of three conventional resin composites in class II restorations. Dent Mater.

[15] Mikhail SS, Schricker SR, Azer SS, Brantley WA, Johnston WM (2013). Optical characteristics of contemporary dental composite resin materials. J Dent.

[16] Ferracane JL (2013). Resin-based composite performance: are there some things we can't predict?. Dent Mater.

[17] Taheri MM, Kadir MR, Shokuhfar T, Hamlekhan A, Shirdar MR, Naghizadeh F (2015). Fluoridated hydroxyapatite nanorods as novel fillers for improving mechanical properties of dental composite: synthesis and application. Mater Des.

[18] Mirjalili A, Zamanian A, Hadavi SM (2019). The effect of TiO2 nanotubes reinforcement on the mechanical properties and wear resistance of silica micro-filled dental composites. J Compos Mater.

[19] Peterson J, Rizk M, Hoch M, Wiegand A (2018). Bonding performance of self-adhesive flowable composites to enamel, dentin and a nano-hybrid composite. Odontology.

[20] Cho K, Rajan G, Farrar P, Prentice L, Prusty BG (2022). Dental resin composites: a review on materials to product realizations. Compos B Eng.

